# Concern about the present status of diabetes & take positive action

**Published:** 2010-11

**Authors:** Yang Wenying

**Affiliations:** Department of Endocrinology, China-Japan Friendship Hospital, Beijing 100029, China ywy_1010@yahoo.com.cn

The global prevalence of type 2 diabetes has shown a trend of rapid growth over the past few decades. According to the National Health and Nutrition Examination Survey, U.S. (NHANES 2005-2006) more than 40 per cent of U.S. adults have diabetes or pre-diabetes^1^. According to statistics of International Diabetes Federation (IDF), two individuals develop diabetes every 10 sec worldwide, and two individuals die of diabetes-related conditions every 10 sec worldwide^2^. Diabetes therefore has become a very serious public health problem with a heavy socio-economic burden to each country. Asia is the world’s most populated area and over 56 per cent of the world’s population lives in the continent^3^. As Asia is hit hardest with diabetes, any subtle changes of diabetes mellitus occurrence can have a great influence on the overall global trends. And the effect of diabetes control in Asia may have an important impact on the global response to diabetes-related stress.

In the context of the general rise of diabetics worldwide, a more rapid growth is seen in the Asian regions. IDF estimates for 2010 point out that six Asian countries are among top 10 countries worldwide in prevalence of diabetes viz., United Arab Emirates (18.7%), Saudi Arabia (16.8%), Bahrain (15.4%) Kuwait (14.6%), Oman (13.4%) and Malaysia (11.6%). The top five Asian countries are: India (50.8 million), China (43.2 million), Pakistan (7.1 million), Japan (7.1 million) and Indonesia (7 million). Bangladesh is expected to replace Japan in 2030 and rank at the 8th4. The results of China National Diabetes and Metabolic Disorders Study (June 2007 to May 2008) showed that among adults over 20 yr of age, the total prevalence rates of pre-diabetes and diabetes [including impaired glucose tolerance (IGT) and impaired fasting glucose (IFG)] were 9.7 and 15.5 per cent and the number of diabetics was estimated to be 92.4 million adults at the age of 20 yr or older and 148.2 million adults would have pre-diabetes^5^. The results were much higher as compared with the previous survey in China, even higher than the IDF predicted value for China in 2030. Considering the fact that the actual prevalence of diabetes is higher than predicted, each country should urgently update epidemiological data to specify present situation of diabetes prevention and control, and make countermeasures according to specific epidemiological characteristics.

National survey in China showed that the prevalence of diabetes had the following characteristics: *(i)* Increased with age; *(ii)* Males had higher prevalence than females, among adults aged 20-60 yr; *(iii)* the prevalence was significantly higher among urban residents than among rural residents (11.4 vs. 8.2%), and the gap in underdeveloped areas was larger. But there was no significant difference in prevalence of pre-diabetes between urban and rural residents (14.9 vs. 16.0%), and the prevalence had been as high as 15.9 and 16.6 per cent between developed and underdeveloped rural areas; *(iv)* There were independent positive correlations between overweight, obesity, central obesity as well as low levels of education and the incidence of diabetes and pre-diabetes^5^. These results reflected epidemic characteristics of diabetes in China to a certain degree. However, with economic development and urbanization, the lifestyle linked changes towards westernization, such as having high-fat, high-caloric diet, and long sedentary hours without physical activities, *etc*. The sharp increase in number of people with obesity and pre-diabetes is due to an imbalance between economic development and education. For example, in urban Indian residents the prevalence of diabetes increased from 13.9 to 18.6 per cent within 6 yr, which is higher than that in the town (16.4%) and peri-urban villages (9.2%)^6^. A survey on South Korea’s rural population showed that the prevalence of diabetes and IGT was 6.9 and 21.9 per cent in 1997, which increased to 11.7 and 38.8 per cent in 2003^7^. Six surveys conducted between 1991 and 2004 in Japan’s Okinawa region showed that the prevalence of diabetes increased from 4.7 per cent in 1991 to 8.4 per cent in 2004. The increase in prevalence of diabetes was higher in men than in women and showed a positive correlation with the proportion of residents who went shopping by car^8^. Even people with the same genetic background, Japanese in the United States had a higher prevalence of diabetes and insulin resistance^9^. The prevalence of diabetes in Hong Kong and Taiwan, where fast economic development started more than a decade ago, was significantly higher than that in the mainland of China for the periods 1985-1994 and 1995-2003^10^. Urbanization, changes in environment and lifestyle therefore have had a crucial effect on the incidence of diabetes.

Ageing population is another problem which people face in the Asian region. Large population and high prevalence of diabetes lead to a rapid increase in the number of aged patients in Asia. For example, Singapore has the fastest growing elderly population in the world. Prevalence of diabetes among people aged 60-69 yr was as high as 32.4 per cent^11^. Qiao *et al*^12^ summarized from 11 studies of 4 countries that prevalence of diabetes in Chinese and Japanese increased with age, and among people aged 70-89 yr it was still at peak.

BMI levels in the Asian population are generally lower than those in Western countries, but the Asian population are still at a higher risk of diabetes^13^. There is evidence to suggest that social changes occur within a surprisingly short period of time, resulting in more significant environmental and lifestyle effects on the population. But do the Asian population also have a genetic susceptibility and unique genetic characteristics? Japanese researchers have been conducting studies to explore type 2 diabetes associated genes^14^,^15^ but more extensive and intensive studies are required to understand the diabetes-related theory in Asia.

The fast rise of economy and large population in Asia lead to a huge increase in diabetics. Facing the challenges, the whole nation need to take immediate action to intervene diabetes among people at high risk effectively, prevent the growth of incidence of diabetes, take multi-factor control of long-term complications in diabetic patients and reduce the damage to health caused by the chronic complications of diabetes.
